# Outpatient revision shoulder arthroplasty can Be safe and effective: a matched analysis

**DOI:** 10.1016/j.jsea.2026.100037

**Published:** 2026-05-27

**Authors:** Asim A. Khan, Zaamin B. Hussain, Ryan M. Lew, Arden C. Shen, Jennifer Kurowicki, Brian Forsythe, Brian J. Cole, Nikhil N. Verma, Gregory P. Nicholson, Grant E. Garrigues

**Affiliations:** a Department of Orthopaedic Surgery, Rush University Medical Center, Chicago, IL, USA; b Department of Orthopaedic Surgery, The Ohio State Wexner Medical Center, Columbus, OH, USA

**Keywords:** Outpatient surgery, Revision, Shoulder, Arthroplasty, Ambulatory surgery center, Complications, Matched cohort, Patient selection

## Abstract

**Background:**

Advances in anesthesia, perioperative pain management, and clearer criteria based on pre-operative risk factors have enabled a shift of primary shoulder arthroplasty to the outpatient setting with acceptable complication rates. However, as the incidence of revision shoulder arthroplasty increases, the safety of outpatient revision shoulder arthroplasty and appropriate indications for same-day discharge remain less clearly defined. The purpose of this study was to evaluate the safety of outpatient revision shoulder arthroplasty and establish pre and intraoperative criteria for appropriate same-day discharge.

**Methods:**

All revision shoulder arthroplasty procedures performed at a single institution between 2015 and 2024 were retrospectively reviewed. Outpatient revision shoulder arthroplasty with ≥3-month follow-up were matched 1:1 with inpatient revision shoulder arthroplasties. Outpatient status was defined as surgeries performed at ambulatory surgery centers (ASCs) or with admission times ≤24 hours. Surgeries were matched by components explanted and implanted, age, and gender. Demographic data, comorbidities, revision surgery subtype, implant details, major/minor complications, readmissions, and patient-reported outcomes were recorded.

**Results:**

A total of 38 surgeries (19 inpatient and 19 outpatient) were retrospectively analyzed. Outpatients (mean age 61.84 ± 7.78 years) included both ASC (n = 9) and hospital-based cases (n = 10). Hospital-based outpatients had longer surgical times (169 vs. 101 minutes; *P* = .025) but similar comorbidities and American Society of Anesthesiologists distribution compared with ASC cases. Baseline characteristics between inpatient and outpatient cohorts were similar except higher smoking prevalence and American Society of Anesthesiologists ≥ III in inpatients (*P* = .021 and *P* = .029). In the outpatient group, 9 surgeries (47%) were full exchanges of all components, while the remainder underwent partial modular revisions (head, glenosphere, or polyethylene). At mean follow-up of 20.8 ± 19.3 months, outpatient patient-reported outcomes improved significantly across nearly all measures. Complications occurred in 9 outpatients (hematoma [5, 26%], stiffness [1, 5%], or persistent pain [3, 16%]), with 1 reoperation, compared to 9 inpatient complications and 3 reoperations. No outpatient readmissions occurred; 2 inpatient readmissions were recorded. Rates of minor and major complications did not significantly differ between cohorts (*P* = .754 and *P* = .625).

**Conclusion:**

In this small, carefully selected cohort, outpatient revision shoulder arthroplasty can be safe and effective with complication profiles comparable to inpatient surgery.

The incidence of primary anatomic and reverse total shoulder arthroplasty (rTSA) procedures is increasing.^3^^,^^5^^,^^17^ Not surprisingly, revision shoulder arthroplasty is also increasing,^4^ primarily for periprosthetic joint infection, component wear or loosening, rotator cuff failure, instability, or periprosthetic fracture.^3^^,^^15^^,^^20^ Revision shoulder arthroplasty generally requires a more challenging exposure, more unpredictable bone quality and quantity, and can result in increased blood loss and a higher risk of neurovascular and other perioperative complications.^14^^,^^15^ Inpatient aftercare offers closer post-operative monitoring, access to advanced pain management, transfusion services, and medical comanagement for medically complex patients, leading it to be historically favored for revision cases.^14^^,^^15^ However, more recently, the quest for value-based health care, improved patient satisfaction and reduced hospital-acquired infections with primary shoulder arthroplasty, has prompted some interest in outpatient revision shoulder arthroplasty.^7^^,^^21^^,^^23^

While outpatient primary total shoulder arthroplasty has become a safe and successful option due to advancements in anesthesia, multimodal pain management, and recovery protocols, the safety and feasibility of outpatient revision shoulder arthroplasty remain uncertain.^11^ Previous studies regarding outpatient revision shoulder arthroplasty have focused on comparing outcomes such as complication rates, readmissions, and reoperations between inpatient and outpatient cohorts using large databases or retrospective reviews.^14^^,^^15^ These studies importantly demonstrate that outpatient revision shoulder arthroplasty can be performed with complication, readmission, and reoperation rates comparable to inpatient surgery, though they relied on large administrative databases and had limited patient-level detail on surgical technique and implant specifics.^14^^,^^15^ These findings apply only to carefully selected and medically optimized patients and have been limited to primary shoulder arthroplasty, not revision procedures.^6^^,^^26^ As such, guidelines around whom may be appropriate candidates have not been well defined.

There remains a lack of clarity about the feasibility for revision shoulder arthroplasty in the outpatient setting and the criteria for appropriate patient selection.^8^^,^^15^^,^^22^^,^^23^ The purpose of this study was to compare short-term safety outcomes between outpatient and inpatient revision shoulder arthroplasty and to describe the clinical and surgical characteristics of cases selected for outpatient management at our institution. We aimed to evaluate whether outpatient revision shoulder arthroplasty, in carefully selected patients, can achieve complication, readmission, and reoperation rates comparable to inpatient revision shoulder arthroplasty and to provide preliminary data that may help inform future outpatient selection guidelines. Based on our experience, we hypothesized that with careful selection and surgical planning, outpatient revision shoulder arthroplasty can be performed safely and effectively.

## Materials and methods

### Patient selection

A retrospective review of all patients who underwent outpatient revision shoulder arthroplasty at a single academic orthopedic institution (Rush University Medical Center) between January 2015 and December 2024 was performed. Institutional review board approval was obtained prior to data collection. “Revision shoulder arthroplasty” was defined as any surgical procedure to exchange, remove, or alter 1 or more components of a prior shoulder arthroplasty (anatomic total shoulder arthroplasty, rTSA, or hemiarthroplasty) using the CPT codes 23473 or 23474. Surgeries were divided into outpatient and inpatient cohorts. Outpatient status was categorized as surgery in an ambulatory surgery center (ASC) or hospital-based outpatient setting with discharge within 24 hours of surgery without formal inpatient admission. Patients requiring a stay longer than 24 hours were included in the inpatient cohort. For Medicare cases performed during years when the procedure codes (CPT 23473 or 23474) were listed on the Center for Medicare & Medicaid Services Inpatient-Only list, patients were classified as inpatient regardless of discharge time; a review of clinical records confirmed that these cases were managed with inpatient-level care and overnight admission, consistent with this classification. The 2 cohorts were case-controlled matched 1:1 based on age, gender, and type of arthroplasty implanted (anatomic total shoulder arthroplasty, rTSA, or hemiarthroplasty) and type of arthroplasty explanted. Lack of pre-operative patient-reported outcomes (PROs) or no follow-up > 3 months were considered exclusion criteria.

### Data collection

We extracted demographic and clinical data for each eligible patient from their medical records. Patient characteristics included age, sex, body mass index (BMI), and comorbidities at the time of revision surgery. The comorbidities included hypertension, diabetes mellitus, respiratory disease, congestive heart failure, cardiovascular diseases, liver or renal disease, rheumatoid arthritis, smoking status (active or previous), sleep apnea (with or without continuous positive airway pressure use), and osteoporosis or osteopenia diagnosis. The American Society of Anesthesiologists (ASA) score classification was recorded for each patient as a general marker of medical fitness. The functional status of the patients was recorded pre-operatively (whether they were capable of living independently) and operative reports and implant records were reviewed for surgical details. The reason(s) for revision were indicated by at least one of the following: glenoid component loosening or wear, humeral component loosening, subscapularis failure, rotator cuff insufficiency, component dissociation, instability (recurrent dislocation), periprosthetic fracture, persistent pain, or periprosthetic joint infection. Indications for revision were not mutually exclusive.

Each revision was categorized as a one-stage or planned two-stage procedure and further classified as a full implant exchange or partial modular component-only exchange, with documentation of all implanted and explanted components. Operative time, estimated blood loss, complications (major and minor), and any deviations from the standard post-operative recovery pathway were recorded. Major complications were defined as adverse events that necessitate substantial medical treatment, such as an unplanned reoperation, hospital readmission for inpatient management, or complications with significant long-term consequences in functional status. Minor complications were defined as those that were conservatively managed and did not require surgical reintervention or hospital admission. Pre-operative and latest follow-up PRO measures were collected to assess clinical efficacy of the revision. It included the American Shoulder and Elbow Surgeons shoulder score and the Patient-Reported Outcomes Measurement Information System (PROMIS) computerized tests for physical function, pain interference, and depression.

### Statistical analysis

Statistical analysis was performed with IBM SPSS Statistics (version 30.0.0.0; Armonk, NY: IBM Corp). Descriptive statistics were used to summarize the demographics, surgical characteristics, and complications. Two-sided exact McNemar tests were used to evaluate binary outcomes and 2-tailed paired *t*-tests were used to compare continuous outcomes. Fisher exact test and Welch *t*-test were used to compare categorical data and continuous variables, respectively, in unmatched independent cohorts. Statistical significance was set as *P* < .05.

## Results

**Figure 1:**
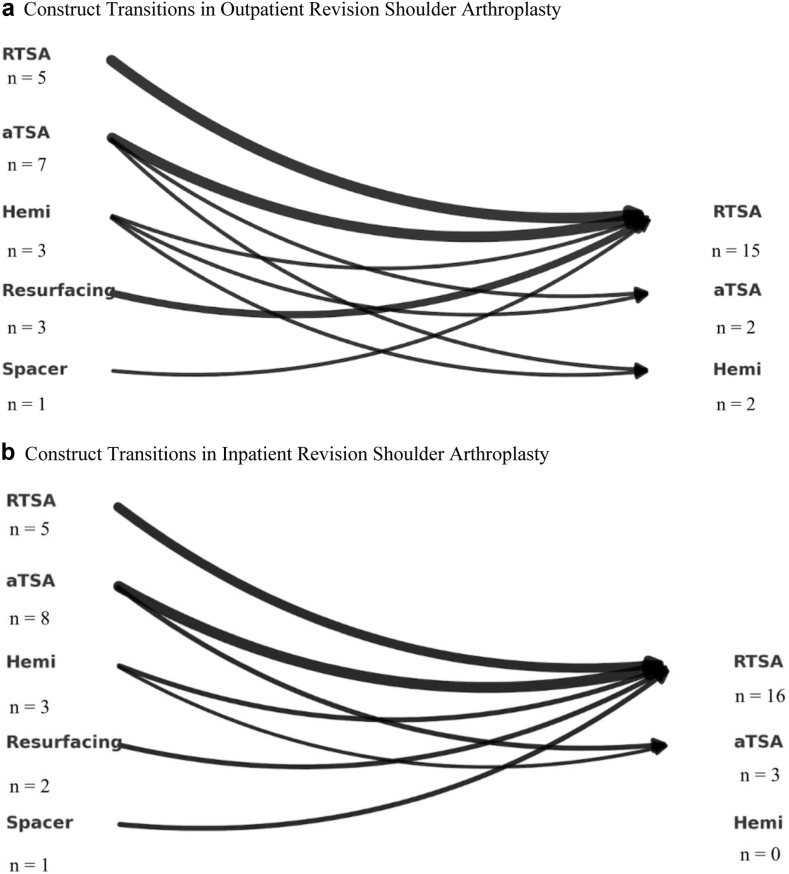
(**a**) Construct transitions in outpatient revision shoulder arthroplasty. (**b**) Construct transitions in inpatient revision shoulder arthroplasty. **(a and b**) Line thickness corresponds to the number of cases undergoing each revision construct transition.

**Figure 2:**
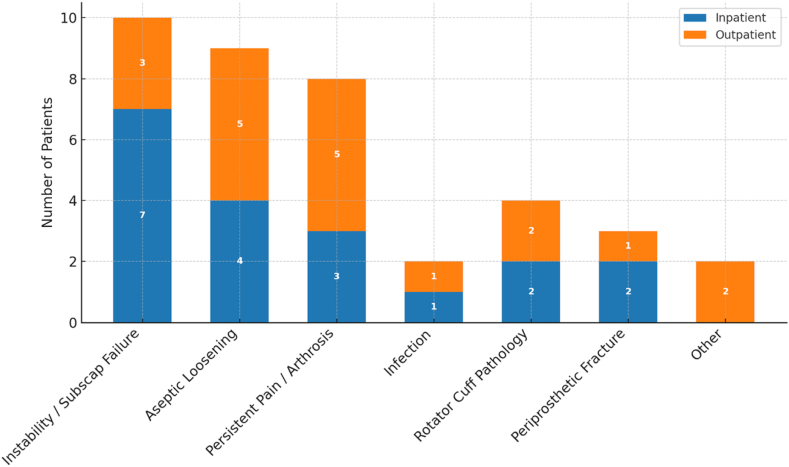
Indications for revision shoulder arthroplasty inpatient vs. outpatient.

Between May 2017 and December 2024 there were 261 surgeries with CPT code 23473 or 23474 performed at our institution, comprising 121 partial or staged revisions (23473) and 140 complete revisions (23474). Nineteen surgeries met our criteria for outpatient status with minimum 3-month follow-up. These were matched 1:1 with those undergoing inpatient surgery based on age, sex, and types of components explanted and implanted, resulting in a total of 38 surgeries included in the final analysis (Fig. 1, *A* and *B*). The 2 cohorts had very similar baseline profiles, with the only significant differences being ASA classifications and smoking status (Table I). The inpatient cohort contained significantly more patients with ASA 3 and had significantly more patients that were either active or previous smokers. The indications for revision surgery in both cohorts are shown in Figure 2. No patients originally scheduled for outpatient revision shoulder arthroplasty required conversion to inpatient admission.

**Table I:** Baseline characteristics and surgical parameters.

Characteristic	Outpatient (n = 19)	Inpatient (n = 19)	*P* Value
Demographics			
Sex (M:F)	13:6	10:9	.289∗
Laterality (R:L)	12:7	12:7	1.00∗
Age, yr, mean ± SD	61.84 ± 7.78	61.0 ± 11.37	.722†
BMI, mean ± SD	29.28 ± 8.11	31.13 ± 7.34	.436†
ASA (1/2/3/4)	2/15/2/0	2/9/8/0	.**029**‡
Surgical parameters			
Location (ASC: hospital)	9:10	NA	NA
Surgical time	137.05 ± 68.5	111.79 ± 26.18	.096†
Estimated blood loss	202.94 ± 165.57	342.11 ± 285.9	.074†
Drain placed	31% (6)	100% (19)	**.0002**∗
Comorbidities			
Smoking (active or previous)	5% (1)	47% (9)	**.021**∗
Functional status (independent)	100% (19)	89% (17)	.500∗
Obesity (BMI >30 kg/mˆ2)	26% (5)	42% (8)	.508∗
Hypertension	42% (8)	47% (9)	1.00∗
Diabetes mellitus	16% (3)	21% (4)	1.00∗
Respiratory disease	5% (1)	0% (0)	1.00∗
Congestive heart failure	0% (0)	5% (1)	1.00∗
Cardiovascular comorbidities	21% (4)	21% (4)	1.00∗
Liver disease	0% (0)	5% (1)	1.00∗
Renal disease	0% (0)	16% (3)	.25∗
Rheumatoid arthritis	5% (1)	5% (1)	1.00∗
Previous revision arthroplasty	16% (3)	11% (2)	.625∗
Taking anticoagulants	21% (4)	26% (5)	1.00∗
Previous PJI diagnosis	11% (2)	0% (0)	.500∗
Sleep apnea	16% (3)	32% (6)	1.00∗
Sleep apnea on CPAP	16% (3)	16% (3)	1.00∗
Osteoporosis/osteopenia	0% (0)	11% (2)	.125∗
Pre-operative PROs			
VAS pain	4.40 ± 2.46	5.94 ± 3.09	.166†
ASES	45.83 ± 17.01	36.69 ± 20.1	.256†
SANE	31.7 ± 28.92	31.7 ± 28.92	.517†
VR-12 physical	37.44 ± 8.96	37.23 ± 8.63	.950†
VR-12 mental	50.69 ± 13.25	52.67 ± 12.54	.684†
PROMIS depression	45.68 ± 8.62	50.53 ± 10.26	.443†
PROMIS pain	60.75 ± 6.94	62.1 ± 9.18	.879†
PROMIS upper extremity	31.92 ± 8.49	28.99 ± 8.69	.216†

*SANE*, Single Assessment Numeric Evaluation; *VAS*, visual analog scale; *PRO*, patient-reported outcome; *ASES*, American Shoulder and Elbow Surgeons; *PROMIS*, Patient-Reported Outcomes Measurement Information System; *BMI*, body mass index; *SD*, standard deviation; *ASA*, American Society of Anesthesiologists; *CPAP*, continuous positive airway pressure; *PJI*, periprosthetic joint infection; *VR-12*, Veterans RAND 12-Item Health Survey.

∗ McNemar test.

† Paired 2-tailed *t*-test.

‡ McNemar-Bowker test.

### Outpatient cohort

The outpatient cohort included 19 surgeries in 18 separate patients. Nine (47%) surgeries were performed in ASCs and 10 (53%) were performed in the hospital setting with discharge within 24 hours. Patient demographics and comorbidities were largely similar between groups. Compared to ASC cases, hospital-based outpatients had longer surgical times (169 vs. 101 minutes; *P* = .025). No other significant differences in demographics, ASA class, blood loss, or comorbidities were observed (Table II). Construct transitions in the outpatient cohort most commonly involved anatomic to rTSA of cases (26%, 5/19) and reverse-to-reverse exchanges in (26%, 5/19) (Fig. 1*A*). Less frequent transitions included conversions from resurfacing (16%, 3/19) or hemiarthroplasty to reverse constructs (Table III). Nine (47%) were full exchanges (all components removed), 3 (17%) were prosthetic head exchanges only, 1 (5%) was a prosthetic head exchange and a glenoid component removal, 1 (5%) was a prosthetic head exchange and a glenoid component implantation, 2 (11%) were glenosphere and poly exchanges, 2 (11%) were poly-only exchanges, and 1 (5%) was the second part of a 2-stage revision. Six patients (32%) were discharged with hemovac drains. Of the 18 single stage revisions, 3 involved stem extractions. Individual patient-level details regarding complications, revision procedures and reoperations for both cohorts are presented in Table IV. One patient was reoperated on for chronic persistent pain, likely unrelated to the shoulder. Eight patients had minor complications. Of these 8 with complications, several experienced more than 1 event. In total, 3 had pain (persistent at ≥ three months post-operative or requiring narcotics), 1 had neuritis, 1 had stiffness (≤110° at ≥3m post-operative), 5 had hematomas, 1 had a superficial infection, and 1 had wound dehiscence (Table V). All PROs (visual analog scale pain, American Shoulder and Elbow Surgeons, Single Assessment Numeric Evaluation, Veterans RAND 12-Item Health Survey physical, Veterans RAND 12-Item Health Survey mental, PROMIS pain, and PROMIS upper extremity) showed significant improvement from pre-operative to post-operative time points (*P* < .05), except PROMIS depression (*P* = .848) (Table VI).

**Table II:** ASC vs. hospital-based outpatient characteristics.

Characteristic	ASC outpatient (n = 9)	Hospital-based outpatient (n = 10)	*P* value
Demographics			
Sex (M:F)	6:3	7:3	1.00†
Laterality (R:L)	6:3	6:4	1.00†
Age, yr, mean ± SD	61.33 ± 7.05	62.30 ± 8.74	.793∗
BMI, mean ± SD	27.14 ± 4.12	31.21 ± 10.37	.275∗
ASA (1/2/3/4)	1/8/0/0	1/7/2/0	.474†
Surgical parameters			
Surgical time	101.22 ± 34.19	169.30 ± 76.85	**.025**∗
Estimated blood loss	164.29 ± 89.97	230.00 ± 203.37	.383∗
Drain placed	11% (1)	50% (5)	.145†
Comorbidities			
Smoking (active or previous)	11% (1)	0% (0)	.474†
Functional status (independent)	100% (9)	100% (10)	1.00†
Obesity (BMI >30 kg/mˆ2)	11% (1)	40% (4)	.303†
Hypertension	33% (3)	50% (5)	.65†
Diabetes mellitus	10% (1)	20% (2)	1.00†
Respiratory disease	0% (0)	10% (1)	1.00†
Cardiovascular comorbidities	11% (1)	30% (3)	.582†
Rheumatoid arthritis	0% (0)	10% (1)	1.00†
Previous revision arthroplasty	22% (2)	10% (1)	.582†
Taking anticoagulants	22% (2)	30% (3)	1.00†
Previous PJI diagnosis	22% (2)	0% (0)	.211†
Sleep apnea	11% (1)	20% (2)	1.00†
Sleep apnea on CPAP	11% (1)	20% (2)	1.00†
Osteoporosis/osteopenia	0% (0)	0% (0)	1.00†

*BMI*, body mass index; *ASA*, American Society of Anesthesiologists; *SD*, standard deviation; *ASC*, ambulatory surgery center; *CPAP*, continuous positive airway pressure; *PJI*, periprosthetic joint infection.

∗ Welch *t*-test.

† Fisher exact *t*-test.

**Table III:** Revision parameters.

Revision type	Outpatient	Inpatient
Single stage	95% (18)	95% (18)
aTSA - aTSA	5% (1)	11% (2)
aTSA – rTSA	26% (5)	32% (6)
aTSA – hemi	5% (1)	0% (0)
rTSA – rTSA	26% (5)	26% (5)
Hemi-aTSA	5% (1)	5% (1)
Hemi-rTSA	5% (1)	11% (2)
Hemi-hemi	5% (1)	0% (0)
Resurfacing - aTSA	0% (0)	0% (0)
Resurfacing - rTSA	16% (3)	11% (2)
1st of 2-stage	0% (0)	0% (0)
2nd of 2-stage	5% (1)	5% (1)
Spacer-rTSA	5% (1)	5% (1)

*aTSA*, anatomic total shoulder arthroplasty; *rTSA*, reverse total shoulder arthroplasty; *hemi*, hemiarthroplasty.

**Table IV:** Individual 90-d complications.

Age (yr)	Gender	Revision procedure	Exchange category	Complication	Reoperation
Outpatient					
56	Male	aTSA → aTSA	Prosthetic head-only exchange	Hematoma	No
49	Male	aTSA → Hemi	Head-only + glenoid removal	Persistent pain	Yes
56	Male	aTSA → rTSA	Stemless full exchange	None	No
59	Male	aTSA → rTSA	Stemless full exchange	Stiffness and neuritis	No
53	Male	aTSA → rTSA	Stemless full exchange	None	No
58	Male	aTSA → rTSA	Stemmed full exchange	Hematoma	No
75	Female	aTSA → rTSA	Head exchange, stem retained	None	No
62	Male	Hemi → aTSA	Head-only + poly Implantation	Hematoma	No
56	Female	Hemi → rTSA	Full exchange	None	No
50	Male	Hemi → Hemi	Prosthetic head exchange	Hematoma, persistent pain	No
73	Male	rTSA → rTSA	Full exchange	None	No
73	Male	rTSA → rTSA	Poly-only exchange	None	No
67	Female	rTSA → rTSA	Glenosphere + poly exchange	Persistent pain	No
71	Female	rTSA → rTSA	Glenosphere + poly exchange	None	No
67	Male	rTSA → rTSA	Poly-only exchange	Hematoma, seroma, wound dehiscence, drainage, fevers	No
61	Male	Resurfacing → rTSA	Full exchange	None	No
63	Female	Resurfacing → rTSA	Full exchange	None	No
60	Female	Resurfacing → rTSA	Full exchange	None	No
66	Male	2-stage revision	Spacer → rTSA	Superficial infection	No
Inpatient					
56	Male	aTSA → aTSA	Stemless full exchange	None	No
47	Male	aTSA → aTSA	Stemmed full exchange	None	No
61	Female	aTSA → rTSA	Stemless full exchange	None	No
64	Male	aTSA → rTSA	Stemless full exchange	None	No
68	Male	aTSA → rTSA	Stemless full exchange	None	No
58	Male	aTSA → rTSA	Stemmed full exchange	None	No
71	Female	aTSA → rTSA	Head exchange, stem retained	None	No
64	Female	aTSA → rTSA	Head exchange, stem retained	None	No
37	Male	Hemi → aTSA	Head-only + poly Implantation	Hematoma, wound dehiscence	No
56	Female	Hemi → rTSA	Full exchange	Hematoma, bleeding requiring transfusion	Yes
60	Female	Hemi → rTSA	Full exchange	Shoulder stiffness, frequent dislocations	Yes
71	Male	rTSA → rTSA	Full exchange	Shoulder Instability	No
56	Female	rTSA → rTSA	Poly-only exchange	Superficial infection, AKI	No
73	Female	rTSA → rTSA	Glenosphere + poly exchange	Shoulder stiffness	No
37	Female	rTSA → rTSA	Glenosphere + poly exchange	None	No
66	Female	rTSA → rTSA	Poly-only exchange	Hematoma, persistent pain, neuritis, deep infection, wound dehiscence	Yes
74	Male	Resurfacing → rTSA	Full exchange	Hypersensitivity pneumonia	No
58	Female	Resurfacing → rTSA	Full exchange	Persistent pain, bleeding requiring transfusion	No
62	Male	2-stage revision	Spacer → rTSA	None	No

*aTSA*, anatomic total shoulder arthroplasty; *rTSA*, reverse total shoulder arthroplasty; *hemi*, hemiarthroplasty; *AKI*, acute kidney injury.

**Table V:** Post-operative complication.

Complications	Outpatient	Inpatient
Follow-up, months, mean ± SD	14.84 ± 19.57 (3-74)	31.89 ± 26.65 (3-79)
Patients with minor post-op complication	42% (8)	32% (6)
Patients with major post-op complication	5% (1)	16% (3)
Pain∗	16% (3)	11% (2)
Instability	0% (0)	5% (1)
Nerve issues/neuritis	5% (1)	5% (1)
Stiffness†	5% (1)	11% (2)
Hematoma	26% (5)	16% (3)
Superficial infection	5% (1)	5% (1)
Deep infection	0% (0)	5% (1)
Wound dehiscence	5% (1)	11% (2)
Pneumonia	0% (0)	5% (1)
Bleed requiring transfusion	0% (0)	11% (2)
Extended hospital stay (≥2 d)	0% (0)	5% (1)
Readmission within 90 d	0% (0)	5% (1)
Readmission ever	0% (0)	11% (2)
Reoperation ever	5% (1)	16% (3)

*Post-op*, post-operative; *SD*, standard deviation.

∗ Persistent pain or pain requiring narcortic use at ≥ 3-mo post-op.

† Forward elevation <110° at ≥ 3-mo post-op.

**Table VI:** Patient reported outcome scores outpatient cohort.

Outcome measure	Pre-op	Post-op	Delta	Significance∗
Follow-up, months, mean ± SD	20.79 ± 19.29 (3-71)			
VAS pain	4.4 ± 2.46	1.79 ± 1.75	−2.38 ± 2.86	**0.001**
ASES	45.83 ± 17.01	76.47 ± 16.44	30.64 ± 19.65	**<.001**
SANE	31.7 ± 28.92	65.75 ± 26.55	32.38 ± 26.92	**<.001**
VR-12 physical	37.44 ± 8.96	49.42 ± 13.42	11.98 ± 10.22	**<.001**
VR-12 mental	50.69 ± 13.25	34.47 ± 14.5	−16.21 ± 17.75	**<.001**
PROMIS depression	45.68 ± 8.62	45.27 ± 8.76	−0.56 ± 10.26	0.848
PROMIS pain	60.75 ± 6.94	51.76 ± 9.25	−9.38 ± 8.26	**<.001**
PROMIS upper extremity	31.92 ± 8.49	38.68 ± 9.56	8.1 ± 9.29	**0.008**

*SANE*, Single Assessment Numeric Evaluation; *VAS*, visual analog scale; *Post-op*, post-operative; *PROMIS*, Patient-Reported Outcomes Measurement Information System; *ASES*, American Shoulder and Elbow Surgeons; *SD*, standard deviation; *VR-12*, Veterans RAND 12-Item Health Survey.

∗ *P* value of 2-tailed t-test between pre-operative and post-operative scores.

### Inpatient cohort

The inpatient cohort included 19 surgeries of 19 separate patients. Construct transitions most frequently involved anatomic to rTSA (32%, 6/19) and reverse-to-reverse exchanges (26%, 5/19) (Fig. 1*B*) with additional cases converting from hemiarthroplasty or resurfacing to reverse constructs (Table III). Eleven surgeries (58%) were full exchanges, 2 (11%) were prosthetic head exchanges only, 1 (5%) was a poly exchange only, 2 (11%) were glenosphere and poly exchanges, 1 (5%) was a prosthetic head exchange with a poly implantation, and 1 (5%) was the second part of a 2-stage revision. All inpatients received hemovac drains at the time of surgery. Of the 18 single stage revisions, 5 involved stem extractions (Table IV). Three patients required reoperations for infection, a periprosthetic fracture as a result of trauma, and glenoid loosening. The following 2 patients were readmitted to the hospital: one within 90 days of their surgery due to confusion and extreme fatigue and the other, a patient that was reoperated on, for incision bleeding. Two patients required a blood transfusion. Six patients had minor complications. Of these 6 patients with complications, several experienced more than 1 event. In total, 2 had pain (persistent at ≥ 3m post-operative or requiring narcotics), 1 had instability, 1 had neuritis, 2 had stiffness (≤110° at ≥3m post-operative), 3 had hematomas, 1 had a superficial infection, 1 had a deep infection, and 2 had wound dehiscence (Table V). All PROs showed significant improvement from pre-operative to post-operative time points (*P* < .05), except PROMIS depression (*P* = .103) (Table VII).

**Table VII:** Patient reported outcome scores for inpatient cohort.

Outcome measure	Pre-op	Post-op	Delta	Significance∗
Follow-up, months, mean ± SD	42.05 ± 22.05 (11-83)			
VAS pain	5.94 ± 3.09	2.44 ± 2.97	−4 ± 2.83	**<.001**
ASES	36.69 ± 20.1	74.06 ± 21.52	39.5 ± 24.63	**<.001**
SANE	31.7 ± 28.92	70.47 ± 30.91	44.21 ± 27.57	**<.001**
VR-12 physical	37.23 ± 8.63	50.12 ± 14.88	12.89 ± 13.23	**<.001**
VR-12 mental	52.67 ± 12.54	41.81 ± 16.62	−10.85 ± 16.45	**0.01**
PROMIS depression	50.53 ± 10.26	45.67 ± 10.97	−0.1 ± 20.04	0.103
PROMIS pain	62.1 ± 9.18	50.05 ± 10.1	−6.99 ± 22.35	**0.005**
PROMIS upper extremity	28.99 ± 8.69	38.6 ± 8.16	11.71 ± 9.24	**0.005**

*SANE*, Single Assessment Numeric Evaluation; *Post-op*, post-operative; *PROMIS*, Patient-Reported Outcomes Measurement Information System; *ASES*, American Shoulder and Elbow Surgeons; *SD*, standard deviation; *VR-12*, Veterans RAND 12-Item Health Survey.

∗ *P* value of 2-tailed *t*-test between preoperative and postoperative scores.

## Discussion

The primary finding of this study was that same-day revision shoulder arthroplasty can be performed safely and effectively in a population of carefully selected patients. Overall complication rates, readmissions and reoperations, estimated blood loss, and operative times were neither statistically nor clinically significant between the 2 cohorts. Despite 1:1 matching by age, sex, and revision construct, the outpatient cohort tended to be characterized by lower-risk baseline profiles and fewer technically demanding procedures, indicating that the surgeons' patient selection inherently favored less complex patients and lower complexity cases. The inpatient cohort had a higher anesthetic risk (greater proportion ASA III), prevalence of smoking, and patients who required humeral stem extraction more frequently. These observed differences suggest that surgeon-driven selection criteria played a meaningful role in determining candidacy for outpatient revision shoulder arthroplasty, though the limited sample size precludes drawing definitive conclusions about optimal patient selection thresholds.

Currently, there is a paucity of literature on outpatient revision shoulder arthroplasty. The current literature supports that revision shoulder arthroplasty performed in the outpatient setting does not lead to a significantly increased complication profile when compared to inpatient revision surgery.^14^^,^^15^ Guareschi et al^14^ evaluated the results from the American College of Surgeons National Surgical Quality Improvement Program database and concluded there were no significant differences in overall rates of complications, readmission or reoperation rates between the outpatient and inpatient rTSA groups. This study evaluated a large database so individual patient-level granularity was not possible to extract. In addition, a study by Hurley et al^15^ reported no significant difference in 90-day complications, readmissions, or reoperations when comparing revision rTSA between outpatient and inpatient settings. In contrast, our matched analysis includes both anatomic and reverse revisions, differentiates ASC from hospital-based outpatient settings, and incorporates PROs.

Although both ASC and hospital-based observation cases are categorized as outpatient, these settings are not equivalent in terms of patient and procedural characteristics. ASCs, which lack immediate access to inpatient services, typically apply stricter selection criteria such as younger patients, lower BMI, fewer comorbidities, and procedures with low anticipated blood loss or shorter operative times.^13^^,^^19^ In contrast, hospital-based outpatient units can accommodate a broader spectrum of patients, including those with higher BMI, more complex revision constructs, or greater anticipated surgical time because resources for escalation such as transfusion or overnight monitoring, are immediately available.^13^^,^^25^ In our cohort, hospital-based outpatients had significantly longer operative times and trended toward higher BMI, consistent with the likely selection criteria applied to these patients, and the previous studies highlighting more complex cases being preferentially performed in the hospital setting where resources allow for escalation if needed.^7^^,^^13^ Comorbidity rates and ASA distribution did not significantly differ, aligning with previous medical literature, and demonstrating that careful patient selection is necessary for outpatient revision arthroplasty in both environments.^16^

Outpatient complications were largely minor and included hematoma, transient neuritis, stiffness, and persistent pain. Only 6 outpatients (31%), with 1 performed ASC, had a drain placed, compared to 100% in the inpatient cohort. Nevertheless, hematoma rates were similar between groups, and drain placement must be weighed against individualized bleeding risk, patient inconvenience for at-home management, and the risk of drain site infection. The inpatient cohort demonstrated a broader range of complications, including higher rates of transfusion, wound dehiscence, and deep infection. This likely reflects greater comorbidity burden, higher ASA classification, and more frequent need for humeral stem extraction, all factors that increase surgical complexity and perioperative risk. Furthermore, only 1 outpatient required reoperation compared to 3 inpatients, demonstrating that outpatient revision shoulder arthroplasty did not carry a disproportionate risk of major adverse events in our cohort. Despite these baseline differences, the complication rates in each cohort lie within a previously published range of revision shoulder arthroplasty, supporting that outpatient revision shoulder arthroplasty, in specific cases, is not associated with a higher observed risk of either minor or major complications when compared to inpatient revision shoulder arthroplasty.^1^ We recommend surgeons should continue to consider individual comorbidities that may predispose the need for hospitalization after revision surgeries as well as the expected surgical tactics needed and perform risk stratification.^12^^,^^22^, ^23^, ^24^

Outpatient revision surgery reduces hospital-associated expenses by minimizing or even eliminating the need for an inpatient hospital stay.^9^^,^^15^ Although prior literature suggests outpatient shoulder arthroplasty reduces hospitalization-related costs, our study did not include direct financial analysis and these claims cannot be directly extrapolated to revision procedures from this data.^5^^,^^10^^,^^12^^,^^18^

Given the economic feasibility of outpatient revision shoulder arthroplasty, establishing clear recommendations for patient selection, perioperative management, and surgical setting is essential to ensure safety and reproducibility. Key criteria for patients to become outpatient candidates include medical stability, absence of uncontrolled comorbidities such as severe cardiopulmonary disease, and adequate social support for post-operative recovery.^23^ In contrast, patients with limited ambulation, congestive heart failure, ASA class III status, advanced age, or active tobacco use require closer consideration for inpatient management due to their higher likelihood of unplanned readmission.^2^^,^^21^ Low risk of blood loss is also an important selection criterion for outpatient revision shoulder arthroplasty and is defined by normal pre-operative hemoglobin levels, an absence of bleeding disorders, and no history of blood transfusion.^2^^,^^24^ Caution should be exercised with patients who have a transfusion history because it is a predictor of adverse events in outpatient arthroplasty. From a surgical point of view, stem extractions should be approached with caution in the outpatient setting, given the association with prolonged operative time and increased blood loss.^10^^,^^27^ However, lower risk humeral component revision may be appropriate for the outpatient setting. This may include revising the following: (1) platform stems (n = 5 in our cohort) that are neither proud nor have male tapers, where the surgeon is confident the stem does not need to be removed either for glenoid exposure and can be retained with acceptable humeral version and tension; and (2) stemless or resurfacing implants (n = 6 in our cohort), where removal is expected to be uncomplicated and iatrogenic humeral bone loss minimal. Any revision involving known or suspected periprosthetic infection should be performed in a hospital-based setting rather than an ASC, as hospital facilities provide necessary resources for intraoperative specimen processing including cell culture, frozen section analysis, and synovial fluid cell counts, which are challenging to obtain in ASC environments.

This study had several limitations. It is a single-institution retrospective series with a modest sample size, increasing the risk of type II error and limiting subgroup analyses. While our analysis found no statistically significant difference in adverse event rates between surgical settings, 1:1 matching by age, sex, and implanted and explanted components cannot fully adjust for the inherent selection bias. Patients with lower medical risk and less anticipated surgical complexity, which likely influenced the observed safety profile, were intentionally selected for outpatient cases. Important unmeasured factors like social support and bone quality were not captured and may have further contributed to case selection. As a result, the between-group comparisons should be interpreted within the context of this selection process. Furthermore, the duration of follow-up differed between cohorts, which may influence detection of later events. Additionally, the outpatient cohort was heterogeneous, comprising 9 ASC-based cases with same-day discharge and 10 hospital-based cases discharged within 24 hours; these 2 settings differ in patient selection thresholds, available resources, and perioperative pathways, and this heterogeneity should be considered when interpreting our findings as representative of a uniform outpatient population. Lastly, formal cost analyses and patient-reported satisfaction assessments were not performed in this study.

Future work should validate these findings in larger, multicenter cohorts with standardized outpatient protocols. Incorporating formal cost-effectiveness analyses and PRO measures will clarify the economic and quality of life benefits of outpatient revision shoulder arthroplasty. Additionally, studies should evaluate perioperative factors such as the role of drain placement and post-operative oral antibiotics, which remain highly variable in practice and lack clear consensus guidelines.^28^^,^^29^ Additionally, this study did not separately analyze hospital-based outcomes for infection-related revisions, and our recommendations intentionally exclude ASC use for any cases with known infection due to limitations in specimen processing capabilities (culture, frozen section, cell count) and the need for potential extended monitoring. The CPT coding challenges (23473 and 23474) and reimbursement barriers that many ASCs face when scheduling revision cases also represent important practical limitations that may affect the generalizability of these findings, though our results suggest that appropriately selected cases can be safely performed when these barriers are overcome. Further research should focus on developing scoring systems that combine medical risk factors with social factors to help surgeons more accurately determine which patients can safely undergo outpatient revision surgery.

## Conclusion

Revision shoulder arthroplasty performed in the outpatient setting can be safe and effective in carefully selected patients, with complication profiles comparable to inpatient surgery. Given the small sample size and retrospective design of this study, future larger, prospective studies are needed to establish evidence-based criteria for outpatient revision shoulder arthroplasty candidacy.

## Disclaimers:

Funding: No funding was disclosed by the authors.

Conflicts of Interest: Grant E. Garrigues reports the following disclosures: Academic Orthopaedic Consortium: board or committee member; Aevumed: stock or stock options; American Shoulder and Elbow Surgeons: board or committee member; Arthroscopy Association of North America: board or committee member; CultivateMD: stock or stock options; DJ Orthopaedics: IP royalties; other financial or material support; paid consultant; paid presenter or speaker; Elsevier: publishing royalties, financial or material support; Genesys: stock or stock options; Journal of Shoulder and Elbow Surgery: editorial or governing board; Mitek: paid consultant; Patient IQ: stock or stock options; Restor3d: paid consultant; stock or stock options; ROM 3: stock or stock options; Sparta Biopharma: stock or stock options; Tornier: IP royalties; paid consultant. Gregory P. Nicholson reports the following disclosures: American Shoulder and Elbow Surgeons: board or committee member; Anika: IP royalties; Arthrosurface: paid presenter or speaker; AzurMed: paid consultant; Innomed: IP royalties; Stryker: IP royalties; paid consultant. Nikhil N. Verma reports the following disclosures: Stryker Corporation: type: other professional activities; discloser type: IP royalties; discloser type: IP Royalties; discloser type: IP royalties; tetrous: type: stock; MLB Team Physician Society: type: Board of Directors or committee member Self. Brian J. Cole, reports the following disclosures: American Journal of Sports Medicine: Editorial or governing board; Arthrex Inc: IP royalties, paid consultant, research support; Elsevier; publishing: IP royalties, publishing royalties, financial or material support; National Institutes of Health (NIAMS and NICHD): research support. Brian Forsythe, reports the following disclosures: American Orthopaedic Society for Sports Medicine: board or committee member. Video Journal of Sports Medicine: Editorial or governing board; reviewer: AJSM, OJSM, Arthroscopy, JBJS, JSES, ISAKOS; Arthrex, Inc: fellowship & research support; Elsevier: publishing royalties, financial or material support; Smith & Nephew: fellowship Support; Conmed Linvatec: fellowship support; Stryker: fellowship support; iBrainTech: stock or stock options; Zuno Medical: stock or stock options; Sparta Biopharma: stock or stock options. Any additional authors, their immediate families, and any research foundations with which they are affiliated have not received any financial payments or other benefits from any commercial entity related to the subject of this article.
